# Management approaches for primary hepatic lymphoma: 10 year institutional experience with comprehensive literature review

**DOI:** 10.3389/fonc.2025.1475118

**Published:** 2025-03-20

**Authors:** Jennifer Ma, Remy Daou, Josiane Bou Eid, Beatrice Fregonese, Joe El-Khoury, N. Ari Wijetunga, Brandon S. Imber, Joachim Yahalom, Carla Hajj

**Affiliations:** ^1^ Department of Radiation Oncology, Memorial Sloan Kettering Cancer Center, New York, NY, United States; ^2^ Department of Family Medicine, Saint Joseph University, Beirut, Lebanon; ^3^ Department of Radiation Oncology, University of North Carolina (UNC) School of Medicine, Chapel Hill, NC, United States; ^4^ Department of Radiation Oncology, Cleveland Clinic, Abu Dhabi, United Arab Emirates

**Keywords:** primary hepatic lymphoma (PHL), diffuse large B cell lymphoma (DLBCL), indolent lymphoma, non-Hodgkin lymphoma (NHL), liver neoplasms, radiation therapy (radiotherapy)

## Abstract

**Purpose/objective:**

Primary hepatic lymphomas (PHL) are an extremely rare form of non-Hodgkin Lymphoma (NHL) for which there are no established treatment guidelines, with available literature largely comprised of small case reports. Therefore, we evaluate our institutional experience treating PHL within the context of existing literature to better understand treatment modalities, role of radiotherapy (RT), and outcomes.

**Materials/methods:**

We conducted a single institutional retrospective study of all patients with PHL diagnosed from 2000-2021, defined as a biopsy-proven liver lesion in the absence of other lymphomatous solid organ involvement, except for concurrently diagnosed hepatosplenic lymphomas. Subgroup analysis was performed for diffuse large B-cell lymphoma (DLBCL) and indolent lymphomas, which included marginal zone lymphoma (MZL), Grade 1-2 follicular lymphoma (FL), and low-grade B-cell lymphoma (BCL), NOS. Univariable (UVA) and multivariable analysis (MVA) for overall survival (OS) were performed using the Cox proportional hazards model. A literature review was conducted using key words “liver”, “lymphoma”, and “treatment” to identify relevant literature.

**Results:**

We identified 30 patients with PHL within the institutional cohort and 192 patients from comprehensive literature review. Subgroup analysis of DLBCL included 15 patients. On MVA for OS, only ECOG score (p=0.02) and Lugano stage (p=0.04) remained significant. Subgroup analysis of the indolent lymphoma group included 9 patients. On MVA for OS, only age remained significant. Systemic therapy was the most common treatment modality overall (20 patients; 67%) with surgery, radiation and observation utilized in 4 patients (13%) each. Seventeen (57%) of patients were alive at the time of data collection, with 8 (27%) deceased and 5 (17%) lost to follow-up.

**Conclusion:**

PHL are an extremely rare subtype of NHL for which there is no clear treatment consensus. Primary hepatic DLBCL appears to be treated mostly with chemotherapy with good disease control. For indolent PHL, low-dose RT appears to have good overall disease control with minimal toxicity. Our RT data is limited by the short duration of follow-up for patients receiving RT compared to those who received chemotherapy, surgery or observation. However, our results are encouraging for the use of RT for appropriate patients with indolent PHL.

## Introduction

Primary hepatic lymphoma (PHL) is extremely rare, and accounts for only 0.4% of all primary extra-nodal non-Hodgkin lymphomas (1). Due to the low prevalence of disease, there are no established treatment guidelines, and the existing literature mostly entails small case series or single case reports.

The treatment approach to PHL is guided essentially by the disease histology, with diffuse large B-cell lymphomas (DLBCL) (2) treated primarily with systemic therapy and indolent lymphomas treated with various modalities including observation, surgery or systemic therapy. There have been limited reports on the use of radiotherapy for PHL in literature.

In a retrospective SEER (Surveillance, Epidemiology, and End Results) database analysis published in 2020, 1372 cases of primary hepatic lymphomas were identified between 1975 and 2016. Among those, DLBCL was the most frequent histology (78.8%), followed by T/NK-cell lymphoma (4.7%), marginal zone lymphoma (MZL; 4.5%), Burkitt (4.3%), follicular (3.1%), and small lymphatic lymphomas (SLL; 2.5%). It was also noted that the annual prevalence of PHL increased with time from 1975 to 2016 (3).

Patients may present with right upper quadrant abdominal pain, nausea or vomiting, as well as the typical B symptoms including fever, weight loss and constitutional symptoms. Older age (80 years or older), male gender, black race, unmarried status, and histology of DLBCL or T/NK-cell lymphoma were associated with a significantly increased risk of cancer-related death (3, 4). A retrospective study conducted through the Rare Cancer Network on 41 patients with PHL found a median survival at 163 months among patients of mixed PHL histology, with a 10-year OS at 59%. Positive prognostic factors include: the presence of fever, the absence of weight loss, and normal hemoglobin level (5). Main characteristics identified among patients with PHL include hepatitis B or C infection, cirrhosis, or concomitant hepatocellular carcinoma (6).

Management of PHL and selection of treatment modalities is challenging given the rarity of PHL and lack of established guidelines.

Therefore, we set out to evaluate our institutional experience treating PHL within the context of existing literature to better understand the treatment modalities, role of radiotherapy (RT), and outcomes. Traditionally, radiotherapy for indolent lymphomas has involved treatment of up to 2400 cGy in 12 fractions, however the use of low-dose radiotherapy of 400 cGy in 1-2 fractions has been increasingly utilized (7). To our knowledge, use of low-dose RT for liver lymphomas has not previously been reported in the literature.

## Materials and methods

We conducted a single institutional retrospective analysis of all patients with PHL diagnosed from 2000-2021. PHL was defined as a biopsy-proven liver lesion in the absence of other active concurrent cancers or lymphomatous solid organ involvement, except for concurrently diagnosed splenic lymphomas. Patients with other cancer diagnoses within 5 years of PHL diagnosis and non-hepatic primary lymphoma were excluded. Subgroup analysis was performed for two histologic subgroups: diffuse large B-cell lymphoma (DLBCL) and indolent lymphomas, which was comprised of marginal zone (MZL), follicular (FL), and low-grade B-cell lymphoma (BCL), NOS. Response was assessed by Deauville criteria with scores of 1-2 considered as complete response (CR) per institutional practice. Univariable (UVA) and multivariable analysis (MVA) for overall survival (OS) were performed for the overall cohort and subgroup analyses using the Cox proportional hazards model and Kaplan Meier curves were generated for overall survival (OS) and progression-free survival (PFS).

Next-generation sequencing (NGS) was performed on select patients as standard of care using the Memorial Sloan Kettering-Integrated Mutation Profiling of Actionable Cancer Targets (MSK-IMPACT) solid tumor clinical assay, which includes up to 505 genes sequenced to a depth of 700X, with germline correction (8, 9). We examined the genetic profiles of available patients in our cohorts for frequency of mutated genes.

In parallel, a literature search was carried out using the PubMed/Medline database for English and French articles and abstracts. There was no date limit applied, and all relevant studies were included. Some studies’ full texts were unavailable online, and we only had access to their abstracts. The keywords and MeSH words used in the search were “Primary hepatic lymphoma” OR “Liver lymphoma” and the histology sub-type: MALT or Mucosa-associated lymphoid tissue, DLBCL or Diffuse large B-cell lymphoma, Follicular lymphoma, Burkitt lymphoma, Mantle cell lymphoma, T-cell lymphoma, and ALCL or Anaplastic large cell lymphoma. A total of 192 cases of primary hepatic lymphoma published in case reports were found and detailed below by lymphoma sub-type. Descriptive analyses were performed on the combined studies by histologic subgroup.

## Results

### Institutional cohort analysis of patients with PHL

We identified 30 patients with PHL within our institutional cohort and 192 patients from comprehensive literature review (**Table 1**). Of our institutional PHL cohort, the predominant histology types included 15 (50%) patients with DLBCL, 5 (17%) MZL, 3 (10%) FL, 4 (13%) CLL/SLL, 2 (7%) T-cell lymphoma, and 1 (3%) had low-grade B-cell lymphoma, NOS.

**Table 1:** Patient demographics of overall cohort and literature review cases.

Variable (n=30)	N (%)	Literature Review (N=192)
Gender		
Male	16 (53)	98 (51)
Female	14 (47)	76 (40)
Unknown	0	18 (9)
Race		
White	25 (84)	N/A
African American	1 (3)	
Asian	4 (13)	
Risk Factors		
Smoking	10 (33)	N/A
Hepatitis C	3 (10)	31 (16)
Hepatitis B	2 (7)	32 (17)
Autoimmune Conditions	4 (13)	16 (8)
Environmental Exposure	2 (7)	N/A
Symptoms at Presentation		
Incidental Finding	9 (30)	47 (24)
Abdominal Pain	8 (27)	60 (31)
Weight Loss	6 (20)	31 (16)
Fatigue	4 (13)	5 (3)
Jaundice	3 (10)	14 (7)
Diarrhea	3 (10)	1 (0.5)
Nausea/Vomiting	3 (10)	3 (2)
Fever	2 (7)	30 (16)
Night Sweats	2 (7)	9 (5)
Median LDH (U/L)	261 (127-2116)	N/A
Median Hemoglobin (g/dl)	12.9 (8.4-15.9)	N/A
Median Platelets	231 (78-432)	N/A
ECOG		
0	25 (83)	N/A
1	3 (10)	
2	2 (7)	
Lugano Staging		
I	25 (83)	187 (97)
III	3 (10)	2 (1)
IV	2 (7)	3 (2)
Bulky Disease	6 (20)	1 (0.5)
Nodal Involvement	3 (10)	5 (3)
Extranodal Sites Spleen	1 (3)	N/A
Treatment Modality		
Systemic Therapy	20 (67)	96 (50)
Surgery	4 (13)	69 (36)
Radiation	4 (13)	10 (5)
Observation	4 (13)	11 (6)
Vital Status		
Alive	17 (57)	97 (51)
Dead	8 (27)	36 (19)
Lost to Follow-up	5 (17)	3 (2)
Median Follow-up (years)	6	2

Overall demographics, disease characteristics and response are noted in **Table 2**. Median follow-up was 6 years. Sixteen patients were male (53%), 25 (84%) were Caucasian, 10 (33%) had a history of smoking, 3 (10%) had prior hepatitis C infection and 2 (7%) had prior hepatitis B infection. PHL was mostly identified incidentally (9 patients; 30%), with 8 (27%) presenting with abdominal pain and 6 (20%) presenting with weight loss. Other presenting symptoms included fatigue (4 patients; 13%), jaundice (3 patients; 10%), diarrhea (3 patients; 10%), and nausea/vomiting (3 patients; 10%). At the time of diagnosis, median LDH was 261 (range 127-2116), median hemoglobin was 12.9 (range 8.4-15.9), and median platelet count was 231 (range 78-432).

**Table 2:** Primary hepatic lymphomas by subtype among institutional cohort and literature review cases.

Histology	Institutional Cohort N (%) (n=30)	Literature Review (n=192)
Diffuse Large B-Cell Lymphoma	15 (50)	76 (40)
- Germinal Center	7 (23)	
- NOS	6 (20)	
- Activated B-Cell	2 (7)	
Burkitt Lymphoma	0	13 (7)
Marginal Zone Lymphoma	5 (17)	66 (34)
Follicular Lymphoma, G1-2	3 (10)	10 (5)
Mantle cell lymphoma	0	2 (1)
CLL/SLL	4 (13)	0
T-Cell Lymphoma	2 (7)	24 (13)
- NOS		16 (8)
- Anaplastic Large T-Cell		9 (5)
Low-Grade B-Cell Lymphoma, NOS	1 (3)	0

Most patients had an ECOG performance status of 0 (25 patients; 83%), and Lugano Stage I (25 patients; 83%), with 3 patients (10%) with Stage III, and 2 (7%) with Stage IV disease. Six (20%) patients had bulky disease measuring ≥7.5cm and 3 (10%) presented with nodal involvement. One (3%) patient had concurrent splenic disease. Systemic therapy was the most common treatment modality overall (20 patients; 67%) with surgery, radiation and observation utilized in 4 patients (13%) each. Seventeen (57%) of patients were alive at the time of data collection, with 8 (27%) deceased and 5 (17%) lost to follow-up. Cause of death for the 8 patients included 5 disease-related deaths and 3 unrelated to PHL. The 5 disease-related deaths included deaths from infection while on immunosuppression after transplant, 1 from infection while receiving systemic therapy, 1 from liver failure, and 1 from widespread progression of disease. The 3 patients with deaths unrelated to PHL included 2 deaths from complications of other unrelated medical comorbidities and 1 patient with no evidence of disease 12 years after last cancer treatment. On univariable analysis, only age (p=0.009; 95% CI 0.030, 0.190) and ECOG score (p=0.048; 95% CI 0.616, 1.000) were found to be significantly associated with overall survival. No variables were found to be significant on multivariable analysis.

In the paired literature review, 96 patients (50%) received systemic therapy, 69 (36%) underwent surgery, 10 (5%) received radiation and 11 (6%) underwent observation (**Table 2**). Seventeen (57%) of patients were alive and 5 (17%) lost to follow-up within our institutional cohort, compared with 97 alive patients (51%) and 3 (2%) lost to follow-up in the paired literature review.

### DLBCL subgroup analysis

We performed a subgroup analysis of DLBCL patients (n=15; **Table 3**), including 7 (44%) with Germinal Center B-cell lymphoma (GCB), 2 (13%) with Activated B-cell Lymphoma (ABC), and 6 (38%) with NOS. Patients were predominantly male (63%) and white (81%). Patients had overall good performance status with 73% of patients with ECOG 0 and 20% of patients with ECOG 1. Lymphomas were generally early stage (73% with Lugano Stage I) including 38% of patients with bulky disease. Almost all patients received systemic therapy at some point (93%) and one patient was observed (7%). Two patients underwent surgery (13%) and none received radiotherapy at any point. ECOG score and Lugano stage were associated with OS on UVA but did not remain significant on MVA.

**Table 3:** DLBCL subgroup analysis for overall survival.

DLBCL Subgroup Variable	MSKCC Cohort (n=15)			Literature Review (n=76)
	N (%)	Hazard Ratio	p-value	N (%)
Median Age	67 (48-89)		0.07	64 (19-82)
Sub-Type	15 (100)			
Germinal Center	7 (44)	6.0	0.06	-
NOS	6 (38)			
Activated B-Cell	2 (13)			
Gender				
Male	10 (63)	0.39	0.78	37 (49)
Female	5 (31)			30 (39)
Race				
White	13 (81)	1.6	0.35	
Asian	2 (13)			
Risk Factors				
Smoking	5 (31)	2.14	0.93	-
Autoimmune Conditions	4 (25)			11 (14)
Environmental Conditions	2 (13)			-
Hepatitis C	1 (6)			11 (14)
Hepatitis B	1 (6)			9 (12)
ECOG				
0	11 (73)	0.68	0.02	-
1	3 (20)			
2	1 (7)			
Lugano Staging				
I	11 (73)	0.71	0.04	73 (96)
II	-			1 (1)
III	2 (13)			2 (3)
IV	2 (13)			
Bulky Disease	6 (38)	0.74	0.91	1 (1)
Nodal Involvement	1 (7)			3 (4)
Extranodal Sites				
Spleen	1			-
Median LDH	304 (127-1598)		0.24	-
Median Hemoglobin	12.8 (8.4-15.9)		0.23	-
Median Platelets	216 (103-503)		0.57	-
Treatment Modality				
Systemic Therapy	14 (93)	0.98	0.12	43 (57)
Surgery	2 (13)	1.02	0.11	21 (28)
Observation	1 (7)			4 (5)
Radiation therapy	-			4 (5)
Response				
Complete Response	12 (80)			31 (41)
Partial Response	1 (7)			3 (4)
Progression of Disease	1 (7)			4 (5)
Lost to Follow-up	1 (7)			2 (3)
Death				12 (16)

Six DLBCL patients with available targeted sequencing data were identified at our institution, with 4 mutations mutated more than once: *TP53* (50%), *MEF2B* (33%), *P2RY8* (33%), and *IRF8* (33%). Pathway mapping of genetic mutations was notable for alterations in cell cycle pathway genes *TP53* and *ATM* (17%). No other pathways had more than one genetic alteration.

### Indolent lymphoma subgroup analysis

**Figure 1:**
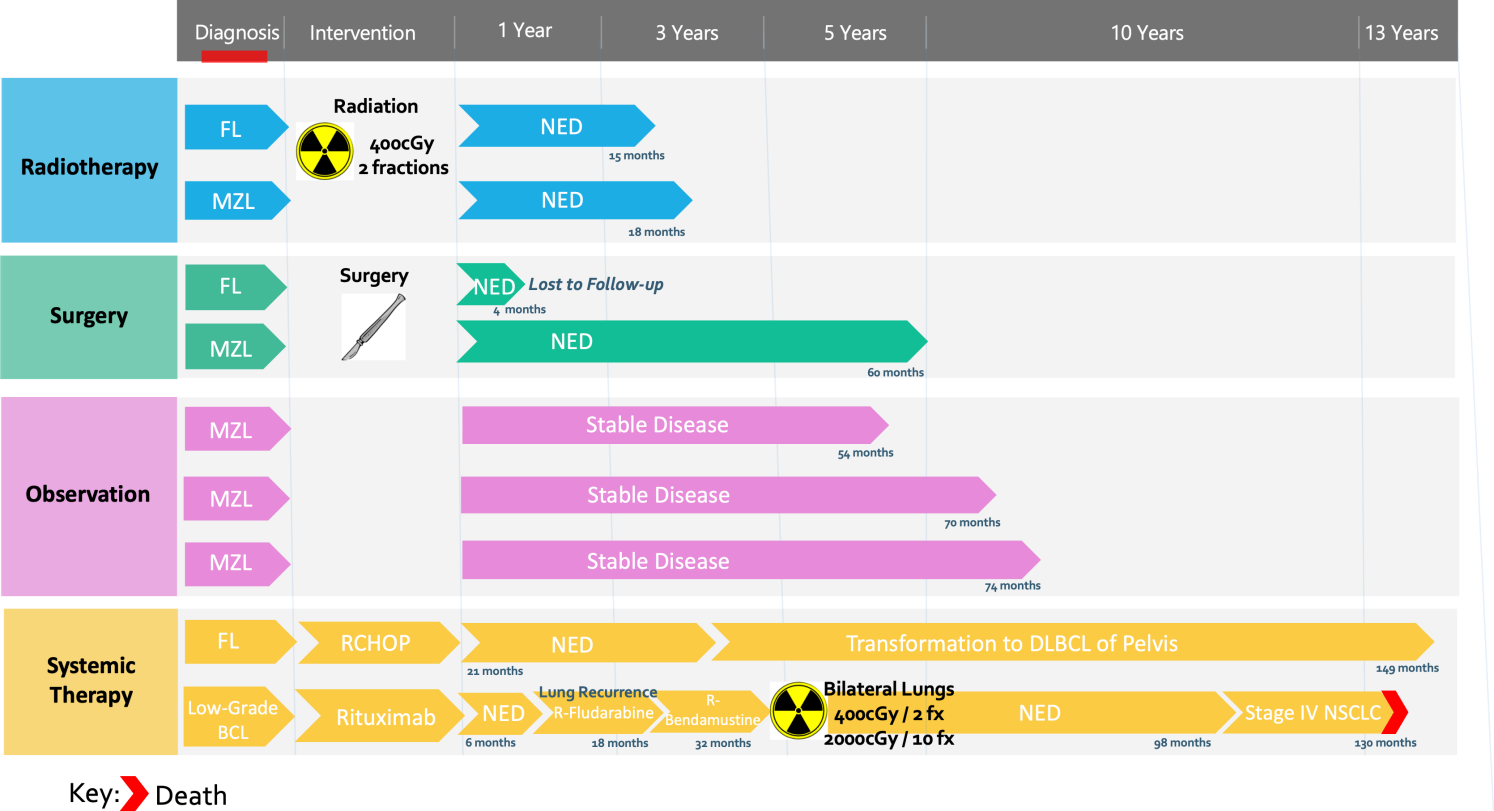
Treatment modalities by patient for indolent PHL cases.

Subgroup analysis of the indolent lymphoma group included a total of 9 patients total, including 5 (56%) with MZL, 3 with FL, and 1 with low-grade BCL, NOS (**Table 4**). Among them, 4 were male, 8 were of white ethnicity, and the mean age was 67 years (range 42-77). All patients were ECOG 0, 89% had Lugano Stage I disease and no patients had bulky disease. Initial treatment modalities were varied including 2 patients each (22%) who received radiation therapy, systemic therapy, surgery or observation (**Figure 1**). Both patients who underwent radiotherapy monotherapy received a low-dose regimen of 4Gy in 2 fractions with complete response and no evidence of disease at 15 and 18 months respectively. Three patients were observed (33%). No patients received combined chemotherapy and radiation as first-line treatment, however one patient received combined chemoradiotherapy as a second-line treatment for lung recurrence. Complete response was seen in 6 patients, progression in 2 patients, and 1 patient was lost to follow-up. Both patients who progressed were initially treated with systemic therapy. Of those, one patient was treated with 6 cycles of R-CHOP with CR, followed by transformation to DLBCL 22 months later for which they received systemic therapy. The other patient was initially treated with rituximab with short-interval recurrence 4 months later for which they received 2 additional systemic therapy regimens as well as radiotherapy to the lungs bilaterally (**Figure 1**).

**Table 4:** Indolent lymphoma subgroup analysis for overall survival.

Indolent Lymphomas Subgroup Variable	MSKCC Cohort (n=9)			Literature Review (n= 78)
	N (%)	Hazard Ratio	p-value	N (%)
Median Age	67 (42-77)		0.03	62 (30-89)
Subtype				
Marginal Zone Lymphoma	5 (56)			66 (85)
Follicular Lymphoma, G1-2	3 (33)			10 (13)
Low-Grade BCL, NOS	1 (11)			-
Mantle cell lymphoma	-			2 (3)
Gender				
Male	4 (44)	1.0	0.22	35 (49)
Female	5 (56)			34 (44)
Race				
White	8 (89)	1.1	0.44	-
Asian	1 (11)			
Risk Factors				
Smoking	3 (33)	1.2	0.19	16 (21)
Hepatitis C	1 (11)			18 (23)
Hepatitis B	1 (11)			2 (3)
Autoimmune Disorder	1 (11)			
ECOG				
0	9 (100)			-
1				
2				
Lugano Staging				
I	8 (89)	0.9	0.17	77 (99)
II	-			1 (1)
III	1 (11)			-
IV	-			-
Bulky Disease	0			-
Nodal Involvement	1 (11)			-
Median LDH	261 (153-296)		0.21	-
Median Hemoglobin	12.9 (11.4-16.7)		0.41	-
Median Platelets	231 (147-447)		0.69	-
Treatment Modality				
Radiation	2 (22)	3.4	0.14	3 (4)
Systemic Therapy	2 (22)	1.0	0.24	30 (38)
Surgery	2 (22)	0.54	0.61	40 (51)
Observation	3 (33)			5 (6)
Response				
Complete Response	6 (66)			40 (51)
Progression	2 (22)			6 (77)
Lost to Follow-up	1 (11)			1 (1)

Median survival in our cohort was 6 years, compared with 2 years among the literature review cohort.

### Literature review

To build upon our institutional experience, we conducted a literature review to contextualize our experience within the broader literature. Existing literature consists largely of small patient cohorts or individual patients with heterogenous treatment approaches based on primary histology. Below we describe the basic demographics, clinical presentations, treatment approaches and outcomes, when available, categorized by lymphoma histology.

### DLBCL subgroup analysis

DLBCL is the predominant histology observed among primary hepatic lymphomas (6, 10). We found 75 cases of primary hepatic DLBCL in 64 studies, with a predominance among male gender (male to female ratio: 1.27), and an mean age of 60.6 years (range 19-82).

Some patients had concomitant cancers including esophageal stromal tumor (11), tumor forming pancreatitis (12) and HCC with gastric adenocarcinoma (13). One patient had a synchronous DLBCL lymphoma in the colon (14). Viral infections such as HCV (13, 15–24), HBV (11, 15, 18, 25–28), HIV (29) and EBV (28, 30, 31) were the most common predisposing factors. Other factors such as auto-immune and hepatic disease were also observed in some cases, including alcoholic cirrhosis (32), primary biliary cirrhosis (33), recurrent hepatic IPT (12), Sjogrens syndrome (33–35), sarcoidosis (36), and polymyositis (30, 37). Five patients received methotrexate for treatment of polyarthritis (27, 28, 36, 38, 39). One patient was noted to have a history of IV drug use (19).

The main clinical presentation varied among patients. Many reported GI symptoms such as right upper quadrant pain (14, 18, 19, 26, 28, 29, 34, 36–38, 40–56), abdominal discomfort (57–60), jaundice (40, 41, 47, 57, 61–63), melena (14), and left upper quadrant pain (64).

Nineteen patients presented with fever (11, 21, 30, 36–40, 46, 48, 50, 51, 53, 63, 65–68), 11 with weight loss (11, 14, 18, 19, 39, 43, 46, 56, 59, 62, 69), one with night sweats (34), and only 4 cases had all B symptoms (29, 50, 53, 60). Other systemic symptoms such as loss of appetite (70) and altered mental status (66, 67) were present. The hepatic mass was incidentally found on imaging for routine follow-up of a chronic disease in 10 cases (13, 17, 23–25, 27, 31, 32, 50, 71), and, in one case, during a workup of a pancreatic mass (12).

On physical examination, one study described a right upper quadrant mass bulge in 7 patients (15) and 2 cases were found to have hepatomegaly (61, 65). One patient presented with generalized edema (72). In 2 studies, patients were diagnosed after presenting with abnormal laboratory results (elevated LFTs (67) and cytopenia (30)).

In 2013, Kashimura M. et al. reported 2 cases where the diagnosis was made on autopsy. Both cases presented initially with fever and elevated LFTs with normal imaging: One was treated with corticosteroids for hypercalcemia, and the other with antibiotics for cholecystitis and peritonitis (67).

First-line treatment strategies were heterogeneous but were mainly chemotherapy-based. Few patients underwent partial hepatectomy alone (11, 15, 43, 52, 73). Methotrexate was discontinued in patients who were receiving it as the only intervention (28, 30), or as adjunct to other treatment such as surgery (27), R-THP-COP (rituximab, pirarubicin, cyclophosphamide, vincristine, and prednisolone) (38), R-CHOP (39), and one month of prednisolone (36).

CHOP regimen was used alone (14, 22), or preceded by 1 cycle of BACOP (bleomycin, driamycin, cyclophosphamide, vincristine, and prednisone) (17) or CEOP (cyclophosphamide, etoposide, vincristine, and prednisone) (23), or in combination with partial hepatectomy (20, 46, 53), or radiation therapy (one patient received 30.6Gy/Fr (65), another received 5x1.8Gy per week with 18Mv IMRT for a total dose of 31Gy (31)). The most commonly used chemotherapy regimen was R-CHOP (13, 15, 25–27, 30, 33, 35, 46, 48, 51, 58, 60, 63, 65). In some cases, it was given prior to resection, without{sp}69 {/sp}or with radiofrequency ablation (44) or radiation therapy (19) (1.5Gy in 4 fractions using a 2 field 3D conformal technique with 10MV photon beam therapy on day 1, 2, 9 and 12). In other instances, it was combined with partial hepatectomy (18, 48, 49), or as part of other regimens. One patient received R-CHOP and was maintained on cyclophosphamide alone for 1 year (58), while another patient received 1 cycle of R-CHOP followed by 5 cycles of R-MegaCHOP and intrathecal chemotherapy (56). Three patients were treated with R-THP-COP alone (55, 61), or with surgery (13). One patient underwent partial hepatectomy followed by COP therapy (60), another received rituximab with bendamustine (70), and one patient was treated with cyclophosphamide and methylprednisolone (59). Only one patient underwent autologous stem cell transplant, for which they received induction COP, R-CHOP then MCE (ranimustine, cyclophosphamide, etoposide) (71).

Six patients were not treated for several reasons: being clinically unstable (33, 68), lost to follow-up (41), patient refusal due to pregnancy (42, 72), and premature death (37).

Patients were followed for an average of 24 months (range 1-80, median: 22). Death occurred in three patients from aplasia and sepsis after R-CHOP (47, 50) or cyclophosphamide and methylprednisone (59). Partial regression was noted in one of the patients who received R-THP-COP (61), and in one of the methotrexate withdrawal group treated with corticoids alone, who experienced complete remission (CR) after R-CHOP regimen (36). An absence of response with tumor progression was reported in one patient treated with R-CHOP alone. However, the patient maintained complete remission for 3 years (25) after receiving second-line R-HyperCVAD (rituximab, cyclophosphamide, vincristine, doxorubicin, methotrexate, cytarabine and dexamethasone)/R-HD MTX ara-C regimen (high dose methotrexate and cytarabine) with radiation therapy (40Gy for 28 fractions). Tumor recurrence was noted in 3 cases. The first patient received R-CHOP and suffered multiple organ failure (45), the second one received autologous stem cell transplant followed by allogeneic bone marrow transplant second-line (71), and the third patient received rituximab and bendamustine and had lymph node involvement. This patient died after receiving R-miniCHOP (rituximab, cyclophosphamide, doxorubicin, vincristine and prednisolone) and 2 cycles of R-EPOCH (etoposide, prednisone, vincristine, cyclophosphamide, and doxorubicin) (70). Some articles did not provide information regarding outcomes (15, 18, 52, 64, 73). The remainder of the described cases were associated with CR (**Table 5**).

**Table 5:** DLBCL literature review summary by treatment modality.

Initial treatment	Treatment characteristics	Outcome	2nd line	Number of patients
Methotrexate discontinuation	Alone	Complete remission	N/A	2
	Surgery	Complete remission	N/A	1
	R-THP-COP	Complete remission	N/A	1
	R-CHOP	Complete remission	N/A	1
	Prednisolone	Partial regression	R-CHOP	1
Autologous stem cell transplant	Induction: COP, R-CHOP then MCE	Complete remission	N/A	1
Chemotherapy-based	CHOP	Complete remission	N/A	1
	CHOP + BACOP	Complete remission	N/A	1
	CHOP + CEOP	Complete remission	N/A	1
	R-CHOP	Complete remission	N/A	12
		Death due to aplasia and sepsis	N/A	2
		Recurrence	R-CHOP (then MOF)	1
			Autologous stem cell transplant (then remission)	1
			R-miniCHOP + R-EPOCH (then death)	1
		Progression	R-HyperCVAD/R-HD MTX ara-C regimen + radiation therapy (40Gy total dose for 28 days)	1
		No outcome	N/A	2
	R-CHOP + Cyclophosphamide as maintenance for 1 year	Complete remission	N/A	1
	R-CHOP + R-MegaCHOP + intrathecal chemotherapy	Complete remission	N/A	1
	R-THP-COP	Complete remission	N/A	1
		Partial regression	N/A	1
	R-bendamustine	Complete remission	N/A	1
	Cyclophosphamide + methylprednisolone	Death of disease	N/A	1
Partial hepatectomy or tumorectomy	Alone	Complete remission No outcome	N/A	5
	R-CHOP	Complete remission No outcome	N/A	1 1
	CHOP	Complete remission	NA	4
	COP	Complete remission	N/A	1
	R-THP-COP	Complete remission	N/A	1
Radiation therapy	Tumorectomy + R-CHOP + RFA	Complete remission	N/A	1
	R-CHOP + 1.5Gy in 4 fractions using a 2 field 3D conformal technique with 10MV photon beam therapy on day 1, 2, 9 and 12	Complete remission	N/A	1
	CHOP + 30.6Gy/Fr	Complete remission	N/A	1
	CHOP + 5x1.8Gy per week 18Mv IMRT with a total dose of 31Gy	Complete remission	N/A	1

### Indolent lymphoma subgroup analysis

#### Follicular lymphoma

Primary hepatic follicular lymphoma is very rare and limited to only 10 cases cited in 9 papers. Patients included 6 women and 4 men and were of advanced age averaging 65.7 years (range 52-85).

Symptoms upon presentation included right upper quadrant pain (74, 75), jaundice (76) or liver failure (77), and elevated LFTs (78). Tumors were incidentally diagnosed on imaging during a follow up for hepatitis C or EBV (79–81), or a work-up for prostate cancer (82). As for comorbidities, some patients had a history of hepatitis B (78) or C (79, 80), EBV (77, 81), Crohn disease (80), and alcoholic cirrhosis (77).

Almost all patients received systemic therapy. Radiation therapy was not used in any patient. Death occurred in four patients. One of them received rituximab (80), the other one received rituximab combined with melfalan (77), and no details were found for the other 2 cases (76). Complete remission was reported in the remaining cases. Among them, one patient received R-CHOP regimen (75), and four patients underwent partial hepatectomy, alone (79) or with chemotherapy (CHOP regimen (78), rituximab – fludarabine –mitoxantone (81)) or with R-CHOP and microwave ablation (74). The average follow-up was 21.5 months (range 1-48, median: 18).

#### MALT lymphoma

Primary hepatic MALT lymphoma is a rare subtype with only 53 papers found and around 70 cases reported in the literature. In fact, according to a retrospective study conducted in 2003 on 180 extragastric MALT lymphomas from 20 institutions, only 3% had a primary located in the liver (83).

In our literature review, there was no gender predominance noted, and the average age at diagnosis was 60 years (range 30-89). The most common forms of presentation included incidental findings from unrelated complaints (84–92), on routine imaging for chronic hepatic disease follow-up (93–110), or during liver transplant evaluation (111–113). Very few patients presented with abdominal pain (unspecified (110, 114, 115), epigastric (116, 117), or in the right upper quadrant (118–120)), nausea (99), or weight loss (99, 110). Some patients had abnormal findings on physical examination (hepatomegaly (115, 120, 121), or ascites (122)), or elevated LFTs (110, 123–127). In a few cases, concomitant hepato-cellular carcinoma (93, 120) was found. In other cases, prior malignancies were present (gastric carcinoma (89, 91), colonic carcinoma (107), hepatic tumor (119), infiltrating duct breast and thyroid papillary carcinoma (96)). We also noted one case with a previously treated gastric MALT lymphoma (117). Among all the cases, associated diseases were present in the majority of the papers, with the main co-factors being chronic hepatitis B (84, 93, 95, 97, 98, 100, 101, 105, 108, 110, 113, 116, 119, 120, 128), chronic hepatitis C (94, 102, 104, 106, 110, 112, 114, 118, 126, 129, 130), and chronic H.pylori gastritis (85, 91, 94, 102, 110, 116, 117, 123, 124, 131). A single case of hepatitis A infection was published in 2002 (89). Six cases of secondary cirrhosis (103, 109, 112, 113, 126, 132) and four cases of primary biliary cholangitis (111, 122, 125, 133) were cited. We found a limited number of cases with other auto-immune or hepatic diseases such as rheumatoid polyarthritis (98), auto-immune hepatitis (99, 109), Castleman syndrome (125), hemochromatosis (103), non-alcoholic steatohepatitis (124), and Buerger disease (85).

While all reported cases were classified as stage IE Ann-Arbor except for 2 cases, which were stage II (87) and IV (110), treatment modalities differed. Mistaken at first for an hepatocellular carcinoma, almost half of the patients underwent partial hepatectomy or tumor resection, alone or with H.pylori eradication (123), hepatitis C treatment (118) or chemotherapy with regimens such as CHOP (105), Rituximab (124), R-CHOP (86, 101, 102, 131), cladribine (131), and bendamustine with Rituximab (126). Patients with advanced cirrhosis who were eligible for liver transplant underwent total hepatectomy (109, 111–113, 132).

In two cases, a combination of antibiotics for H.pylori eradication and R-CHOP was used (94, 116). Chemotherapy alone (110) was the treatment of choice in some patients, with regimens including Rituximab (88, 99), R-CHOP (95, 119, 127, 131), R-CVP (110), R-THP-COP (122) and Etoposide (133).

Two patients were treated with radiation therapy (110, 121), with one receiving a total dose of 41.4 Gy (19.8 Gy to the entire liver and an additional 21.6 Gy to the tumor (121). One patient received Rituximab and underwent percutaneous radiofrequency ablation (RFA) (134).

Four patients did not receive treatment due to patient refusal or physician’s preference. The first case showed progression after 12 months following Rituximab (99), the second progressed after 32 months (100), the third died from sepsis one month later (117), and there was no mention of follow-up or outcome for the fourth case (114).

As for the two cases with advanced disease, both achieved CR after the first treatment, 6 cycles of chemotherapy for stage IVA lymphoma (110) and partial hepatectomy for the stage II patient (87).

Patients were followed for an average of 23 months (range 1-96) after completion of first-line treatment with a median follow-up of 16 months. Almost all patients achieved CR after first-line treatment. Two deaths were caused by surgical complications (132), and one death occurred due to fulminant hepatitis B and D 3 months after R-CHOP treatment (127).

Recurrence of disease was reported in the following cases: 51 months after a treatment with cladribine followed by R-CHOP{sp}131 {/sp}as a second line, 3 years after R-CVP plus 40 Gy radiation therapy (110), 18 months after R-THP-COP and Rituximab as salvage therapy (122), 14 months after resection followed by Rituximab for 1 month (128), and 30 months after partial hepatectomy with radiation therapy and chemotherapy (98).

Two patients were lost to follow-up (103, 132), and approximately half of the studies did not mention follow-up duration of the patients (**Table 6**).

**Table 6:** MALT lymphoma literature review summary by treatment modality.

Treatment modality	Adjuvant	Outcome	2nd line	Number of patients
**Partial hepatectomy**	None	Complete remission	N/A	18
		Recurrence	Radiation therapy and chemotherapy	1
		Recurrence	Rituximab	1
	H.pylori eradication	Complete remission	N/A	1
	Hepatitis C treatment	Complete remission	N/A	1
	CHOP	Complete remission	N/A	1
	Rituximab	Complete remission	N/A	1
	R-CHOP	Complete remission	N/A	4
	Cladribine	Recurrence	R-CHOP	1
	R-Bendamustine	Complete remission	N/A	1
**Total hepatectomy**	None	Complete remission	N/A	4
		Death due to surgical complications	N/A	1
**Chemotherapy**	No available data	Complete remission (stage IVa)	N/A	1
	Rituximab	Complete remission	N/A	2
	R-CHOP	Complete remission	N/A	3
		Death due to fulminant hepatitis B and D	N/A	1
	R-CVP	Recurrence	40 Gy of radiation therapy	1
	R-THP-COP	Recurrence	Rituximab	1
	Etoposide	Complete remission	N/A	1
	R-CHOP + H.pylori eradication	Complete remission	N/A	2
**Radiation therapy**	No details available	Complete remission	N/A	1
	Total dose 4140 cGy (1980 cGy on entire liver + 2160 cGy on tumor area)	Complete remission	N/A	1
**RFA**	Percutaneous RFA + Rituximab	Complete remission	N/A	1

### Other subtypes of lymphoma

#### Burkitt lymphoma

Only thirteen cases were found in the literature. Half of the patients were females, and the majority were less than 60 years of age except for one 75-year-old patient (mean age 38.5 years). The patients had past medical history of viral infections [EBV (135, 136), HCV (136, 137), HBV (138–140), HIV (135, 140–143)], and one had a history of kidney transplant (136).

The chief complaints were mainly gastrointestinal such as weakness with vomiting and diarrhea (136), nausea (144, 145), abdominal mass (145), and jaundice (140, 142). Abdominal discomfort and pain (135, 139–141, 144, 146, 147), and hepatomegaly (135, 136, 139, 142, 144, 146, 147) were among the most common presentations. B symptoms such as fever (141, 142, 146), night sweats (140), and weight loss (145, 147) were reported in a few patients.

As for treatment and outcomes, two patients underwent surgery with chemotherapy (138) based on hyper-CVAD with methotrexate-Ara-C THP and Rituximab. Death from septic shock was reported in 3 patients who received chemotherapy (141, 143, 145), including a patient who received cyclophosphamide-prednisone-methotrexate-dexamethasone (136). The other cases were treated with regimens including the m-BACOD protocol (methotrexate, leucovorin, bleomycin, doxorubicin, cyclophosphamide, vincristine, and dexamethasone) resulting in tumor lysis syndrome (142), rituximab with cytarabine and dexamethasone with complete remission (135), CHOP with methotrexate and intrathecal cytosine arabinoside and methotrexate with cure (139), and LMB-96 protocol (three doses of high-dose methotrexate, 8 g/m2/dose, and eight doses of HD cytarabine, 3 g/m2/dose) with remission (144). One patient refused treatment and was lost to follow-up (140).

#### Mantle cell lymphoma

Two cases were found in the literature.

The first paper described a 54-year-old man, known to have a history of HBV infection, who presented with weight loss, abdominal distention and hepatomegaly. After treatment with chlorambucil, partial remission was reported with a follow-up of 47 months (148). The second article reported the detailed clinical and pathological presentation of a primary hepatic lymphoma in a 73-year-old woman. Therapies used were not specified for this patient (149).

#### T-cell lymphoma

Sixteen cases on T-cell hepatic lymphomas were found in the literature. Patients included three women and 13 men with a mean age of 48 years (range 30 to 71 years). Chief complaints were either related to liver disease such as jaundice (150, 151), hepatomegaly (151–153), right upper quadrant pain (150, 154, 155) or systemic symptoms such as fatigue (156), edema (151), B symptoms (150, 152, 154, 155, 157, 158). Two patients presented with cytopenia (155, 159) and one patient with toxic hepatitis (158). History of viral infection such as EBV (150, 157), HAV (155), HBV (158, 160), and HCV{sp}153 {/sp}were noted in some cases. One paper cited two patients suffering from chronic alcoholism and chronic drug use (155). Two patients had cirrhosis secondary to hepatitis (153, 160), and one patient suffered from systemic lupus erythematosus (159).

The primary hepatic T-cell lymphomas were almost all treated with chemotherapy based on the CHOP regimen, either alone (151, 155, 158, 160) or adjuvant therapy after surgery (156, 161) coupled with radiation therapy (154, 159) (45 Gy in 15 fractions) (154) or with another chemotherapy regimen (DHAP) (155). One patient received only 20 mg of oral prednisone as short course therapy followed by 5 mg as maintenance (152). Most patients achieved complete remission except for two. One of them progressed after surgery and then treatment was withdrawn (156), and the other received ECHAP as a second-line treatment then died (158). As for the rest of the cases, all ended in death of the patient including esophageal hemorrhage following radiation and chemotherapy in one patient (159), and two patients who received supportive therapy only and died a few days after diagnosis (155, 158). One patient underwent partial hepatectomy and died from post-operative complications (153). Average follow-up was 23 months, with a maximum of 7 years (median: 12 months).

#### Anaplastic large cell lymphoma

Nine cases were found in the literature. Patients included two women and seven men with a mean age of 47 years (range 33-62). Five of the patients had underlying liver disease associated with the following conditions: HIV (162), HIV and HCV (163), HBV and chronic alcoholism (164), celiac disease (165), hereditary hemochromatosis and cirrhosis (166).

The chief complaints included RUQ pain (148, 162, 163, 165–167) or one of the B symptoms such as fever (148, 162–165), weight loss (148, 162–166) or night sweats (166–168). Complete remission after 20 months of follow-up was noted in one patient who underwent hepatectomy followed by 4 cycles of CHOP (165). Partial remission was reported in another patient after 6 cycles of CHOP and etoposide (167). Diagnosis was made on autopsy (163, 164)in two patients who died within a few days of symptom onset. In one patient who experienced hepatic failure, death occurred after a treatment of 60mg of corticosteroid per day (166). Two patients underwent resection of the tumor followed by chemotherapy. In the first case, death occurred 15 months after 6 cycles of CHOP (168). The second patient showed progression after receiving IMVP16 (ifosfamide, methotrexate, and VP-16) and CHOP and autologous peripheral blood stem cell transplantation after induction with ESHAP (etoposide, methylprednisolone, high-dose cytarabine and cisplatin) and CCEP (cyclophosphamide, lomustine, etoposide and prednisolone). Death was reported 3.5 years after surgery (148). Therapies used were not specified in two papers (162, 169).

## Discussion

PHL are a rare subtype of NHL without clear treatment consensus. The treatment approach is driven by the treatment paradigms of the primary lymphoma tumor histology; however, our histology-based literature review demonstrates that treatment approaches are heterogenous and appropriately guided by tumor histology.

DLBCL is the most common subtype of GI lymphomas and is predominantly treated with chemotherapy in both our institutional experience as well as reported literature, with reasonable disease control. This is consistent with the established treatment paradigm for DLBCL due to its more aggressive and diffuse nature (170).

The approach to treatment for indolent PHL is less well established, with various modalities employed in both our institutional cohort as well as the literature review. Although there are limited case reports regarding the use of standard doses of radiotherapy of 30-41.4 Gy for PHL, to our knowledge this is the first case series reporting low dose radiotherapy for the treatment of PHL. Low-dose radiotherapy has been shown to have good local control in select patients with indolent lymphomas (2). At our institution, we use an adaptive approach when treating patients with indolent lymphomas. Appropriate patients are treated with low dose radiotherapy upfront of 4Gy. The option to receive additional radiation is based on PET-guided response assessment 3 months post-RT. Although our cohort data is limited by short follow-up duration, and a very limited number of the patients receiving RT, the data is concordant with local control outcomes of other indolent lymphoma sites.

Indolent lymphomas are slow growing and can often be observed for many months or years before requiring intervention. In our cohort, 33% of patients required intervention beyond observation whereas literature review revealed that all 8 cases of indolent lymphomas were treated with systemic therapy, and none were observed or treated with local therapies such as surgery or radiotherapy. Therefore, our institutional experience is encouraging for the consideration of low dose radiotherapy following an adaptive approach in select patients for effective local control and minimal toxicity, as an alternative to observation, surgery, or systemic therapy.

## Conclusion

PHL are an extremely rare subtype of NHL for which there is no clear treatment consensus. Primary hepatic DLBCL appears to be treated mostly with chemotherapy with good disease control. For indolent PHL, low-dose RT appears to have good overall disease control with minimal toxicity. Our results are encouraging for the use of RT for appropriate patients with indolent PHL, using the adaptive approach of 400 cGy in 1-2 fractions, with the option to receive an additional 20Gy based on PET-guided response assessment 3 months post-RT. Despite the limited number of patients reported in our institutional experience, these promising results should prompt more extensive studies on the use of low dose radiation therapy in indolent PHL.

## Data Availability

The raw data supporting the conclusions of this article will be made available by the authors, without undue reservation.
